# Scholar Plot: Design and Evaluation of an Information Interface for Faculty Research Performance

**DOI:** 10.3389/frma.2019.00006

**Published:** 2020-01-28

**Authors:** Dinesh Majeti, Ergun Akleman, Mohammed Emtiaz Ahmed, Alexander M. Petersen, Brian Uzzi, Ioannis Pavlidis

**Affiliations:** ^1^Computational Physiology Laboratory, University of Houston, Houston, TX, United States; ^2^Visualization Department, Texas A&M University, College Station, TX, United States; ^3^Department of Management of Complex Systems, UC Merced, Merced, CA, United States; ^4^Kellogg School of Management, Northwestern University, Evanston, IL, United States

**Keywords:** information visualization, science of science, scientometrics, research career evaluation, university evaluation

## Abstract

The ability to objectively assess academic performance is critical to rewarding academic merit, charting academic policy, and promoting science. Quintessential to performing these functions is first the ability to collect valid and current data through increasingly automated online interfaces. Moreover, it is crucial to remove disciplinary and other biases from these data, presenting them in ways that support insightful analysis at various levels. Existing systems are lacking in some of these respects. Here we present Scholar Plot (SP), an interface that harvests bibliographic and research funding data from online sources. SP addresses systematic biases in the collected data through nominal and normalized metrics. Eventually, SP combines synergistically these metrics in a plot form for expert appraisal, and an iconic form for broader consumption. SP's plot and iconic forms are scalable, representing equally well individual scholars and their academic units, thus contributing to consistent ranking practices across the university organizational structure. In order to appreciate the design principles underlying SP, in particular the informativeness of nominal vs. normalized metrics, we also present the results of an evaluation survey taken by senior faculty (*n* = 28) with significant promotion and tenure assessment experience.

## 1. Introduction

A growing body of work on academic performance measures has been taking place in the field of research evaluation (Van Noorden, 2010). While grounded in information science, additional efforts have strived to make these metrics broadly available via web-based interfaces to a wide array of academic users. These interfaces are textual, visual, or a combination thereof. Technology/publishing companies play a protagonistic role in these developments, having fielded popular systems, such as Google Scholar (by Google) and SciVal (by Elsevier) (Vardell et al., 2011). Google Scholar is a free bibliographic system, mostly textual in nature, which provides all-encompassing citation data, but requires setup by individual scholars. SciVal is a fee-based system that fashions dozens of academic performance metrics in textual and plot form; it does not depend on individual scholar setup, but provides only a fraction of the citations in Google Scholar.

Academic research groups also advanced interesting scientometric measures and applications. In some cases the focus was on the extraction and presentation of bibliometric data, with emphasis on citations (Plaisant et al., 2008; Robecke et al., 2011; Zhao et al., 2013). In other cases the focus was on interfacing author network information (Tang et al., 2008; Kaur et al., 2014).

A distinct line of research focused on visualization interfaces for scientometric data (Scharnhorst, 1998; MacKenzie, 2009; Nakazawa et al., 2015; Latif and Beck, 2019). In general, visualization has been used to help people understand complex datasets, supporting actionable insights (Yi et al., 2008). Because scientometric data are voluminous and serve an evaluative purpose, visualization in plot and iconic form could be of great utility. Regarding the iconic form, we find the flower metaphor particularly interesting for structural and aesthetic reasons. Flower depictions were used in several data domains. For example, flowers were used as metadata representations of web documents (Chau, 2011) and for the visualization of facebook data (Wang et al., 2015). We also believe that garden metaphors with flowers as key elements, are uniquely suited to represent human ecosystems, such as the academic ecosystem. A garden design (Xiong and Donath, 1999) was used for the visualization of interactions among users in an online environment.

**Figure 1:**
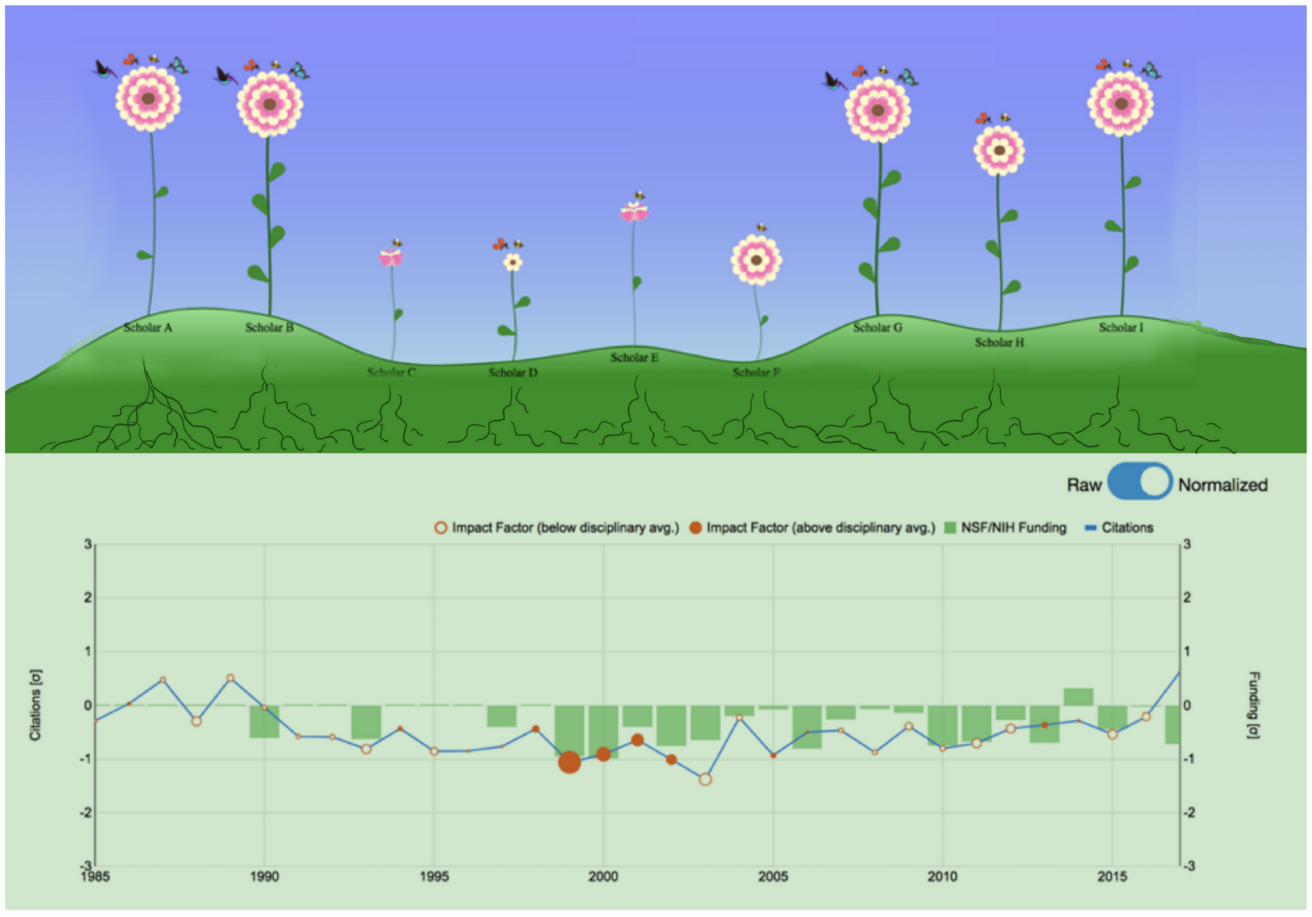
The two faces of Scholar Plot. **(Top)** Iconic representation of individual faculty merit in a computer science department in the U.S. - summer 2018. **(Bottom)** Temporal plot representation of cumulative academic merit for the same department.

Here, we describe the design and evaluation of an innovative scientometric interface that focuses on the research performance of faculty. We call this design Scholar Plot (SP) and we developed a pilot implementation for it, which is accessible at http://scholarplot.org. SP is based on a model that combines three inter-related elements of scholarship. These elements include the ability to raise research funds, and how effectively these funds are used for the production of competitive and impactful publications. By contrast, many scientometric interfaces are not based on an explicit model, presenting either an isolated performance measure (e.g., citations) or a smorgasbord of measures with little apparent connection. Importantly, SP expresses its model in both plot and iconic forms (Figure 1) that naturally scale up from individual faculty to academic departments. Typical scientometric interfaces lack such scalability and multi-modality in the service of a merit model. This points to a key difference of SP from notable recent work designed to communicate insightful details of author personas (cf. Latif and Beck, 2019). SP is not about details and does not focus exclusively on authorship. SP is a visual summary of research grantsmanship, publication competitiveness, and publication impact—three institutional preoccupations of academic systems rooted in research funding.

The intended application of SP is informational. It can be a useful awareness tool for faculty and Ph.D. students. It can also provide a supplementary perspective to promotion and tenure (P&T) committees. In whatever capacity SP is used, is not meant to replace peer judgment or expert opinion; at best, it is hoped to enhance them. Although we believe that performance data and metrics can play a constructive adjunct role in academia, we are against some disturbing trends that seek to deify quantitative evaluations. In this respect, we espouse that the use of SP and other similar systems should be guided by the principles laid out in the DORA declaration (Bladek, 2014) and the Leiden manifesto (Hicks et al., 2015).

## 2. Presentation of the Online Tool

### 2.1. Interface Design

The key aim of SP is to complement the conventional academic CV, enhancing its insightful interpretation. Additional aims of SP are packaging academic profiles for broader consumption and scaling-up certain merit criteria to groups. Accordingly, the design of SP's interface strives to:

- **Design Aim 1**. Bring together some key merit factors, in a way that enhances thoughtful evaluation. Conventional academic CVs contain relevant data, but lack quantification, factor correlation, and visual representation.
- **Design Aim 2**. Convey merit factors in visual forms that appeal to different user groups, ranging from scholars to the public. As academic research and its products are increasingly internitized, alternative impact and broader consumption of science precipitate. For example, altmetrics (https://www.altmetric.com) quantify press and social media activity around scientific articles. Altmetric scores of articles are visualized as color coded donuts, which have become ubiquitous academic badges. For the faculty who create these scientific articles, however, there is no visualization scheme to popularize their scholarly profile.
- **Design Aim 3**. Construct visualizations of merit factors that are organizationally scalable (i.e., faculty → departments). More than other organizations, academia is the sum of its parts - reputable faculty make for reputable departments, which could be viewed as composite scholars. Hence, individual merit criteria that test for reputability (citations) and its contributing elements (funding and publication competitiveness) need to percolate up the organizational units, and fuse.

### 2.2. Design Elements—Model of Merit Factors

Generally, merit criteria are rooted in research scholarship, teaching performance, and service to the scientific community (Chait, 2009); thus, they include broader considerations than the ones targeted by SP. In Ph.D. granting institutions, however, criteria related to research scholarship of faculty weigh the most. Interestingly, different disciplines, and countries construe somewhat differently these criteria. For example, in humanities departments there is emphasis in the production of monographs, while in Science, Technology, Engineering, and Medicine (STEM) departments there is emphasis in the production of peer-reviewed papers. Depending on the science funding system and culture of each country, there are also differences in the importance assigned to the award of research grants.

The merit criteria of relevance to SP focus on the research profiles of STEM faculty in the United States and other similar academic systems. In this context, three key factors are considered, which are often discussed during P&T deliberations (Nir and Zilberstein-Levy, 2006): (a) The ability to raise funds in support of a research agenda. (b) The ability to produce competitive publications. (c) The ability to produce impactful publications. To some degree, funding is a necessary but not sufficient condition, for the production of quality and competitive publications. Hence, what a merit factor representation scheme needs to show is how effectively scholars translate funding to competitive and useful research. Importantly, a merit factor scheme needs to be objective. Several interfaces for academic performance measures fail in this respect. They tend to focus on citations (Federico et al., 2017), presenting them in raw counts, information that is only partly informative and usually skewed.

SP juxtaposes all three merit factors, revealing their interrelationship. SP also presents these factors in both raw and normalized forms for nuanced evaluation. As a raw proxy for funding, SP uses the dollar amounts of grants, credited in the year they were awarded; as a proxy for competitiveness, SP uses the Impact Factor (IF) of the journals where the publications appeared; and, as a proxy for impact, SP uses the papers' citation counts.

Among the said factors and their measures, the competitiveness factor with its IF metric bear further discussion. There has been a long-standing debate about publication merit, which in the past many scholars tautologized with citation impact (Lehmann et al., 2006). Thanks to recent work, however, there is increasing realization that publication merit does not always coincide with citation impact. For example, highly novel works with little grounding in the existing literature are often slow to accrue citations (Uzzi et al., 2013). Irrespective of impact, novelty is a virtue in the research world. Novel concepts that are well argued are likely to appear in top journals, which build their branding not only on impactful but also on novel content.

It is not easy to have papers accepted in prestigious journals. Anecdotally, all academics know that such journals tend to be selective, which adds to their desirability. Unfortunately, not all journals regularly report their acceptance rates and when they do, the released numbers are not independently verified. Nearly all journals, however, receive every year an IF score from the Clarivate Analytics—Journal Citations Reports. Academics are very familiar with IF scores, which by definition measure the number of times selected articles in a journal are cited the last few years. Interestingly, based on existing samples, journal IF scores tend to inversely correlate with acceptance rates (Sugimoto et al., 2013). Hence, we can practically use IF in a different capacity, that is, to index the level of competition papers have to overcome to get published in the corresponding journals.

It is true that a portion of the papers that appear in highly competitive journals will prove useful, attaining high citation counts in due course; the remainder will not, but this does not detract from their original achievement. At the same time, it is also true that some impactful papers get published in moderately competitive venues. Hence, to capture all these nuances, SP employs both a citation metric, akin to eventual usefulness and an IF metric, akin to initial competitiveness of published works. These considerations are often discussed in the context of P&T evaluation (Nir and Zilberstein-Levy, 2006) - thus, their quantification and visualization in SP can play a broadly supportive role.

To address systematic temporal and disciplinary biases, SP normalizes the raw P&T factor values by computing the following z-scores:

- **Normalization of Funding -** *f*^*F*^. The deviation of the scholar's awarded funding in year *t* from the mean awarded funding the same year to scholars belonging in the same discipline.
- **Normalization of Competitiveness -** *f*^*Q*^. The deviation of the scholar's mean IF for journal papers s/he published in year *t* from the mean IF of journal papers published in year *t* by scholars in the same discipline.
- **Normalization of Citations -** *f*^*C*^. The deviation of the scholar's citations for papers s/he published in year *t* from the mean citations accrued by papers published in year *t* by scholars in the same discipline (Lundberg, 2007).

The normalization formula for computing these z-scores is:

(1) $$\begin{array}{l} z_{f^{i}}=\left[\text{ln}\left(1+f_{s,d,t}^{i}\right)-\mu_{d,t}\right]/\sigma_{d,t}, \end{array}$$

where $f_{s,d,t}^{i}$ is one of the three P&T factors (*i* = *F* or *Q* or *C*) for scholar *s*, in discipline *d*, in year *t*; it applies to composite scholars (e.g., departments), too. Note that μ_*d,t*_ is the mean and σ_*d,t*_ is the standard deviation of the underlying disciplinary- and time-specific distribution of $\text{ln}\left(1+f_{s,d,t}^{i}\right)$ values; we apply logarithmic transformation to address the right skewness in the distribution of nominal $f_{s,d,t}^{i}$ values; we add 1 to each individual nominal value to address the singularity encountered for ln(0) (Petersen et al., 2018). Discipline *d* is defined according to the Classification of Instructional Programs (CIP) codes (Morgan et al., 1991) for the departments in the SP database. Academia's mindset remains department-centric and academics draw inferences from cases in similar departments nationwide. The said normalization scheme simply captures this reality. From the bibliometric point of view, such normalization also addresses a difficulty in citation analysis that arises from varying citation rates across disciplines of different size (Petersen et al., 2018).

### 2.3. Multiform Design—Expert vs. Broad Use

#### 2.3.1. Plot Forms for Experts

**Figure 2:**
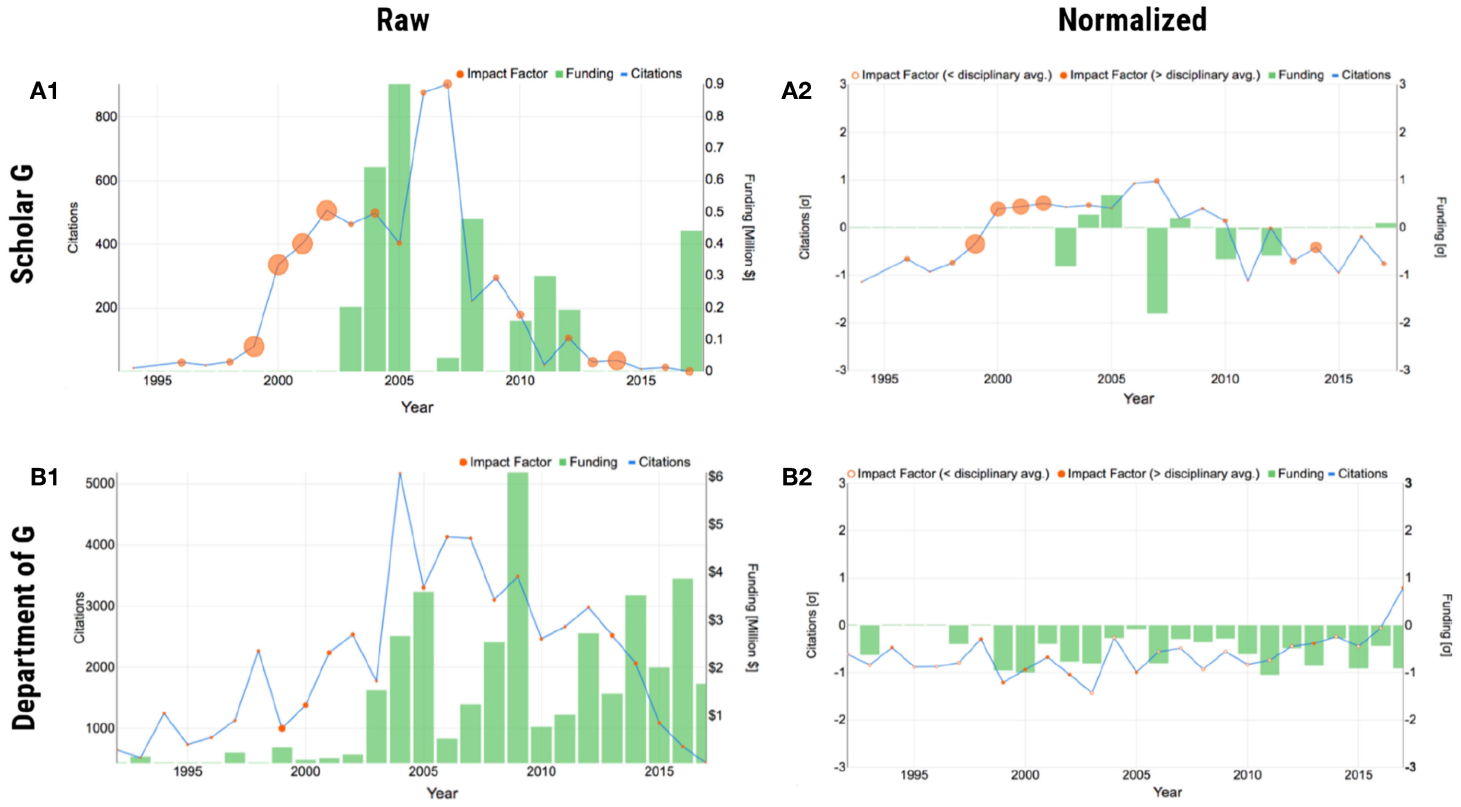
Plot form of SP using scholar G and his department as examples. Scholar G is faculty in the SP database, anonymized for presentation purposes. **(A1)** Raw individual scholar G. **(A2)** Normalized individual scholar G. **(B1)** Raw department of scholar G. **(B2)** Normalized department of scholar G. In the normalized plots, negative z-scores for IF are expressed as hollow disks.

SP primarily aims to enhance faculty CVs and situate them in the departmental and disciplinary context. For this expert level use of SP, we have designed the plot form (Figure 2) that combines time-series representations for the three merit factors. Specifically, total funding per year is given as a bar graph; total citations per year are given as a polyline; mean IF per year commensurates with the size of disks that connect the polyline segments. SP's plot form comes in two versions - the raw plot and the normalized plot. These versions are meant to complement each other in facilitating insightful evaluation, in concert with the information provided in a faculty's CV and other merit review documents.

##### 2.3.1.1. Raw plot

The raw plot makes apparent how the three merit factors interact with each other in the course of the faculty's career, providing also a quantitative sense. For example, the raw plot of scholar G in Figure 2A1 shows that competitive and impactful research the scholar performed in 2000–2002, led to raising research funds in 2003–2005, which in turn led to more impactful work in 2005–2007. It would be very challenging to glean and articulate such information from a conventional academic CV.

##### 2.3.1.2. Normalized plot

The raw plot has two issues—it gives a skewed impression of the scholar's recent citation impact and conveys no information about how the scholar's evolution fares against his/her disciplinary cohort. The latter is often a source of misunderstanding in college level P&T committees, whose members are coming from different departments. The normalized plot addresses both the aforementioned issues, by applying the data transformation given in Equation (1). Figure 2A2 shows the normalized plot, where the temporal and disciplinary biases were ameliorated, giving a more informed picture of the scholar's record. In each year on record, the plot shows the scholar's deviation from the national standard as a *z* score; the national standard (*z* = 0) is defined as the logarithmically corrected mean disciplinary performance the said year for the said factor.

#### 2.3.2. Iconic Forms for Broad Use

There are occasions where a more summative impression of a scholar's record is in order. Such occasions include the annotation of short biosketches, promotional campaigns for fund-raising purposes, and press releases. Summarization can be achieved by collapsing the time-series aspect into a cumulative picture, and by reducing the levels of detail in this picture. SP molds this summary picture, aimed for broad consumption, into the shape of a flower.

We need a living entity as a visual metaphor for scholarly records. The reason is simple - academic records evolve and the iconic model would need to naturally accommodate such an evolution. Rocks for example do not grow, but trees do. The chosen living entity should neatly decompose into three main parts that would correspond to the three merit factors under consideration. The challenge is that one of the merit factors (i.e., citations) alludes to “popularity,” which is not an inherent feature of living organisms. To address this problem we focus on living organisms that have symbiotic relationships with other species, and the two can be depicted together. Then, we can use the symbiotic species attracted to the living organism as a “popularity” factor index.

##### 2.3.2.1. Flower

**Figure 3:**
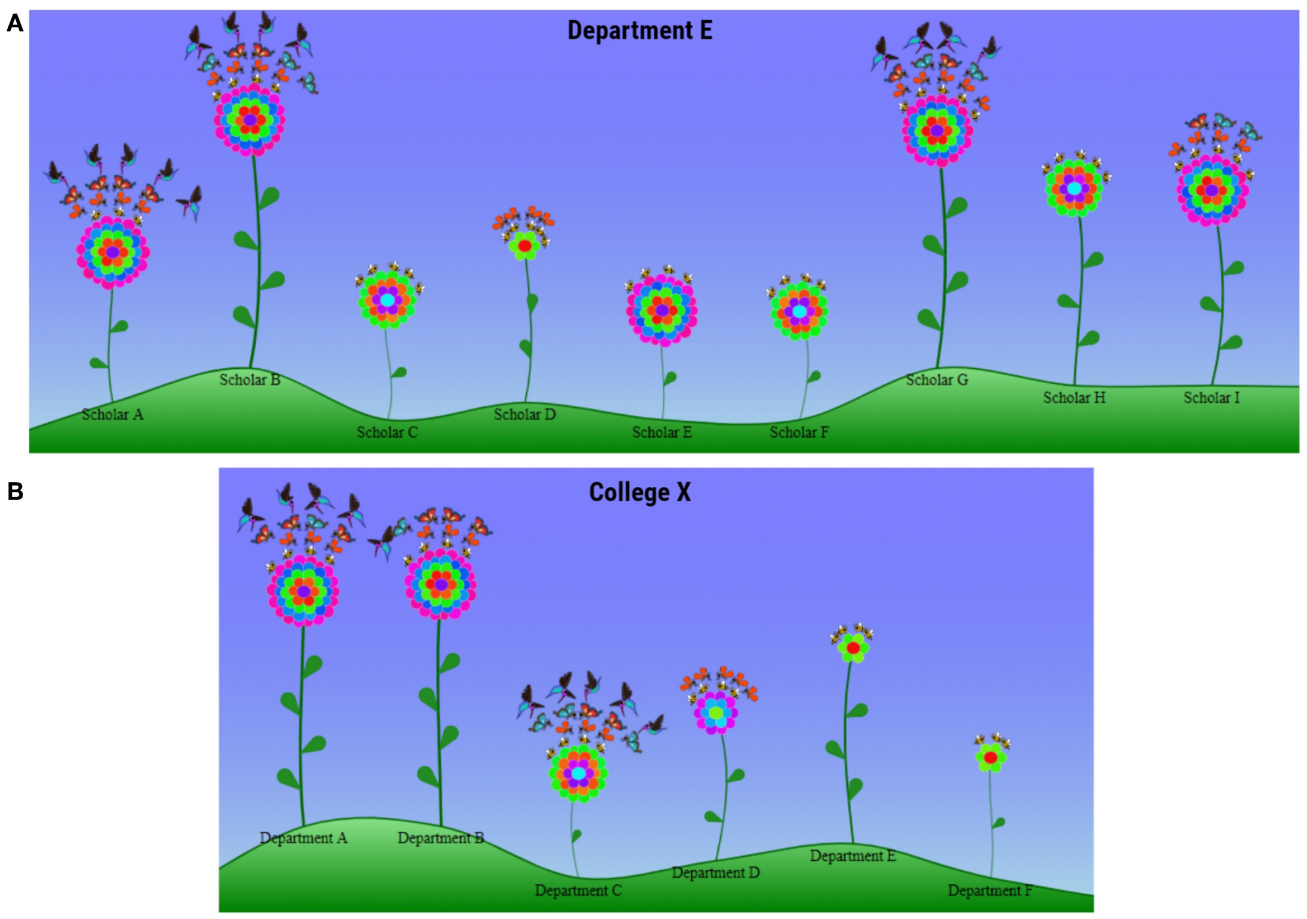
Iconic form of SP. **(A)** Individual faculty of “Department E.” **(B)** Departments of “College X.” Placement of the flowers on the rolling landscape is largely random, to support naturalness; so is the number of pollinators in each layer.

Taken all these considerations into account, the flower, with pollinators as its symbiotic species, is an apt metaphor for the P&T summary model (Figure 3). The flower's leaves correspond to levels of funding; its rounds of petals correspond to publication competitiveness, as expressed by IF; the layers of pollinators the flower attracts correspond to accrued citations. The quantifiable detail drops from the precise numbers depicted in the plot form to quartiles: four leaves, four rounds of petals, and four layers of pollinators correspond to the 4th quartile in terms of funding, IF, and citations for a scholar. Smaller numbers of leaves, rounds of petals, and layers of pollinators correspond to lesser quartiles. The quartiles in this pictorial information can be drawn from the set of faculty within the department or from the national set of faculty belonging to departments with the same CIP code (Figure 3A).

The flower design is consistent with well-established construction principles for computer icons (Huang et al., 2002). Determination of the quantities the flower symbol represents, depends on enumeration of some key constituent elements rather than the apparent size of floral structures; e.g., three leaves for 3rd quartile of funding or two pollinators for 2nd quartile of citation impact. On the one hand, this approach facilitates fast and unambiguous interpretation of the visual metaphor. On the other hand, it releases the designer to impart naturalness to flowers by semi-randomly varying the length of their stems, without any adverse effects on symbolism.

##### 2.3.2.2. Garden

Flowers are fitting visual metaphors for summarizing academic records - their apparent parts and symbiotic species correspond neatly to the three merit factors. Moreover, flowers are the main units of gardens, and in turn, gardens can be used as visual metaphors for academic ecosystems. In the current version of SP we use simple gardens made out of a collection of flowers to depict faculty profiles in departments. Future versions of SP will be enriched with flowerbeds within departments to depict Ph.D. groups led by faculty, and landscaping to convey the quality of the departmental infrastructure.

### 2.4. Scalable Design—Scholars to Organizations

SP visualizes three merit criteria that weigh to one degree or another in academic careers. The same merit criteria also weigh in the research ranking of departments. Hence, the representation forms for persons need to scale to academic organizational units. In interfacial terms, this means that the organizational representations should remain as clear as the individual representations, despite the incorporation of multiple entities. Both the plot (Figures 2B1,B2) and iconic (Figure 3B) forms of SP scale up to departmental representations, because they treat departmental entities as “composite scholars.”

## 3. Data, Evaluation Methods, and Results

### 3.1. Data Sources

The current pilot implementation of SP draws from faculty lists in Ph.D. granting universities in the United States; it also uses funding (NIH/NSF RePORTER tables), bibliometric (Google Scholar), and journal IF (Clarivate Analytics–Journal Citations Reports) data. In general, there are significant challenges in assembling datasets for evaluation of research careers (Petersen et al., 2018). In the case of SP, we had to address the following issues:

- **Faculty Coverage**. Presently, the SP faculty data do not cover Ph.D. granting universities outside the United States. To understand the reason for this limitation, one needs to appreciate that normalization of P&T criteria in SP critically depends on the accuracy of faculty membership in departments. For example, the citations of papers published by faculty *F*_*i*_, belonging to a department with CIP code C, in year *t* are normalized against the citations of all papers published in year *t* by faculty belonging to departments with the same CIP code (e.g., C= Computer Science). Unfortunately, there are no publicly available databases of faculty lists in the United States and elsewhere in the world. Hence, we undertook a lengthy and expensive effort to create such a database for STEM departments in the United States by visiting their People web pages and recording their faculty members. Quality control was performed by repeating this data collection for three consecutive years (2016, 2017, 2018) and reconciling differences. As of summer 2019, the database features 17,491 faculty from 1,656 departments in 143 U.S. institutions. Although this is far from a comprehensive global faculty database, it is well more than a toy dataset. Hence, it provides an excellent base for piloting the design concepts and research metrics outlined in this paper.
- **Funding Coverage**. The funding data do not cover all sources of research appropriations in the United States. Here again there are limitations with the public availability of data. The federal RePORTER has grant tables for some key government funding agencies in the United States, but not for all. Not to mention that funding tables from foundations and corporate sources are totally absent. SP uses funding data from the National Institutes of Health (NIH) and the National Science Foundation (NSF). NIH and NSF are the biggest and most prestigious funding agencies in the United States, which also happen to have the most complete and up to date public funding tables. Importantly, based on literature reports (Petersen et al., 2018), NIH and NSF funding predict career impact, which makes them relevant for piloting the concepts introduced in this paper.
- **Competitiveness Metrics**. The current SP scheme uses only journal IF as a paper competitiveness metric. In some engineering fields, however, faculty publish frequently in refereed conferences. Presently, SP does not include such papers into competitiveness computations, because the corresponding conference proceedings lack impact factor. In a future SP enhancement, conference ranking could be used as a competitiveness indicator for such publications (CORE Conference Portal: http://portal.core.edu.au/conf-ranks/), although this metric is not as well established as the journal IF metric.
- **Name Disambiguation**. The manually harvested faculty database, includes faculty name entries, linked to departmental affiliations and Google Scholar profiles. These faculty names need to be matched to the investigator names listed in the NIH and NSF grant tables, in order to acquire the corresponding award records. There are occasional name conflict issues in this matching. These conflicts stem primarily from inconsistent use of middle initials or middle names. We apply the validated name disambiguation algorithm we reported in our *Science Advances* paper (Petersen et al., 2018), to resolve such conflicts.

### 3.2. Evaluation Survey

To evaluate the usefulness and usability of SP, as well as receive user feedback, we constructed a relevant online survey. The survey is available via SurveyGizmo: https://www.surveygizmo.com/s3/4251954/Scholar-Plot-Survey. We disseminated this survey to *N* = 60 senior faculty with P&T evaluation experience, belonging to six different university departments in the United States. We received completed surveys from *n* = 28 professors in computing, physical sciences, and psychology from universities in Texas, California, and Minnesota. The survey was instructing participants to complete the questions in one uninterrupted session, as their response times were evaluative criteria and breaks would have introduced ambiguities. We gave considerable thought to the design of the survey, which consists of three segments: (a) plot survey; (b) iconic survey; and, (c) perceptions survey. In the next three sections, we detail the survey design of each segment and present the corresponding results. Please note that all cases used in the survey questions are real records from the SP database, which have been anonymized.

#### 3.2.1. Plot Form of SP—Survey Design and Measurements

**Figure 4:**
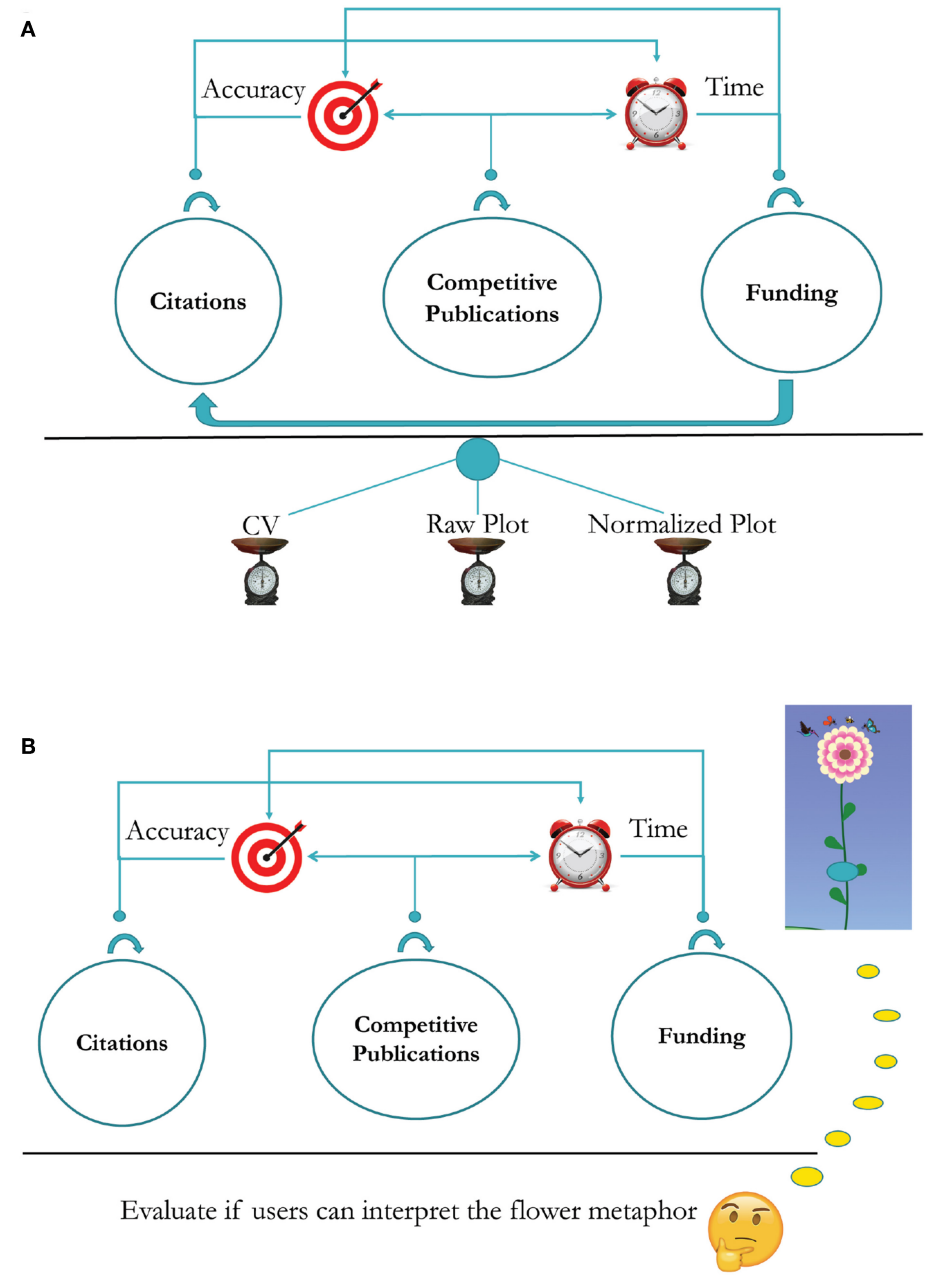
**(A)** Survey design for the plot form of SP. **(B)** Survey design for the iconic form of SP.

Figure 4A outlines the survey design for the plot form of SP. This part of the survey was meant to examine the accuracy and efficiency with which SP users were able to evaluate the citation, publication, and funding record of scholars. The plot survey also examined if SP can facilitate inferences across the funding and citation factors. Because a key aim of the SP interface is to complement the conventional form of academic appraisal, that is, the scholars' CVs, the survey was structured in a progressive manner. First, the respondents had to make evaluative decisions with respect to the three merit factors (i.e., citations, competitiveness, and funding) by looking at a faculty's CV. To standardize this phase of the survey, we chose the Google Scholar profile of the said faculty as his/her academic CV. Hence, the academic CV in this survey is restricted to a verifiable bibliographic section and does not contain other *ad-hoc* sections, such as teaching and service records, which are outside the scope of SP. Next, the respondents had to make a fresh round of evaluative decisions based solely on the Raw Plot of the said faculty. Last, the respondents had to make evaluative decisions anew based this time on the said faculty's Normalized Plot. This order of modality presentation, regarding the faculty's academic record, meant to bring to the fore any corrections or additional insights afforded by the SP plots.

Within each modal session (i.e., CV, Raw Plot, and Normalized Plot), the merit factor questions were also presented in a progressive order. First, the respondents had to answer questions with respect to the chosen scholar's citation record, second with respect to the said scholar's publication competitiveness, and third with respect to the said scholar's funding. In the plot sessions, the respondents were also asked at the end to attempt a cross-factor inference, that is, how funding affected the said scholar's citation record. The order of questions mirrors the priorities of P&T committees in the United States; they tend to value citation impact very highly, as this is akin to scholarly reputation and shapes departmental ranking.

Participants did not receive any instructions prior to the CV section, as they were experienced academic evaluators familiar with conventional forms of academic resumes, such as Google Scholar profiles. Because participants were not familiar with SP, however, the survey had short tutorials prior to the SP Raw Plot and SP Normalized Plot sections. The tutorials consisted of an example plot and a half page description of the plot's features. These tutorials were the only preparation participants had prior to start using SP to answer questions in the corresponding survey section.

To avoid confounding due to disciplinary bias, the sample scholarly record given in the survey was drawn from the discipline of the respondent; for example, if the respondent were a computer science faculty, then the survey was presenting the questions against the record of a computer science scholar; if the respondent was a physics faculty, then the survey was presenting the questions against the record of a physics scholar. To facilitate consistency, these records were drawn from the third citation quartile of U.S. professors in the respective discipline.

**Figure 5:**
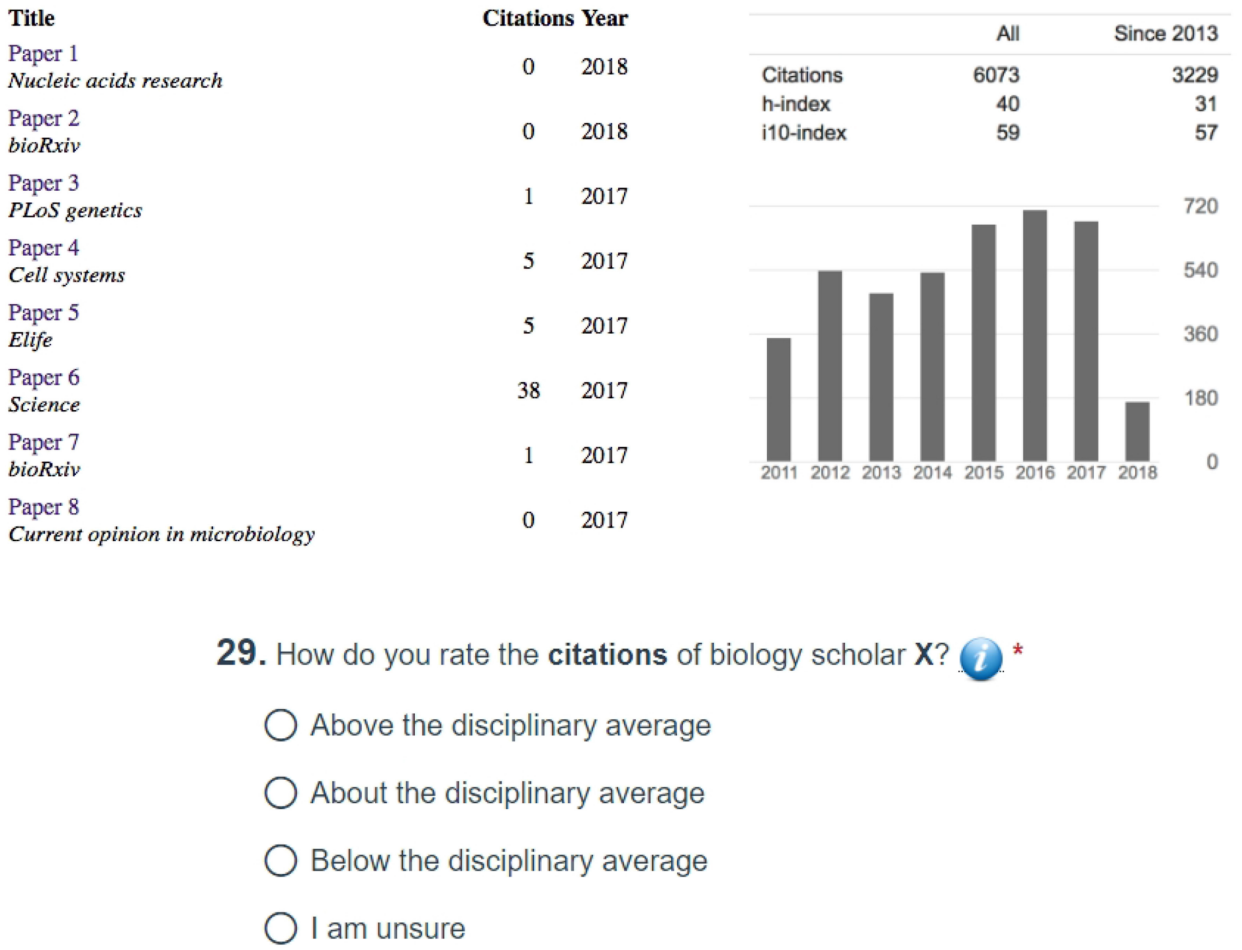
Anonymized Google Scholar profile serving as the basis of comparison with the Raw and Normalized SP forms, when the survey is taken by respondents in Biology Departments. The publications are listed in reverse chronological order, and the respondents have to scroll down to see the full profile of the scholar under examination.

Figure 5 shows the anonymized Google Scholar profile used when the survey was taken by respondents in Biology Departments; the specific question depicted in the figure tests the respondents' understanding of the citation impact for the scholar under review. All the questions across modalities and factors were framed in a similar manner, that is, the respondents were asked to evaluate if the scholar under examination is below, at, or above the disciplinary average in U.S. academia. This aspect of the survey design captures the mindset reflected on typical P&T questions posed to outside referees, such as: “Would you confer tenure [or promotion] to this candidate if s/he were in your department?” The answer to this type of questions trends positive, if the referee considers the candidate to be above the national average; it is unpredictable if the referee considers the candidate to be average; and, it trends negative if the referee considers the candidate to be below the national average.

**Figure 6:**
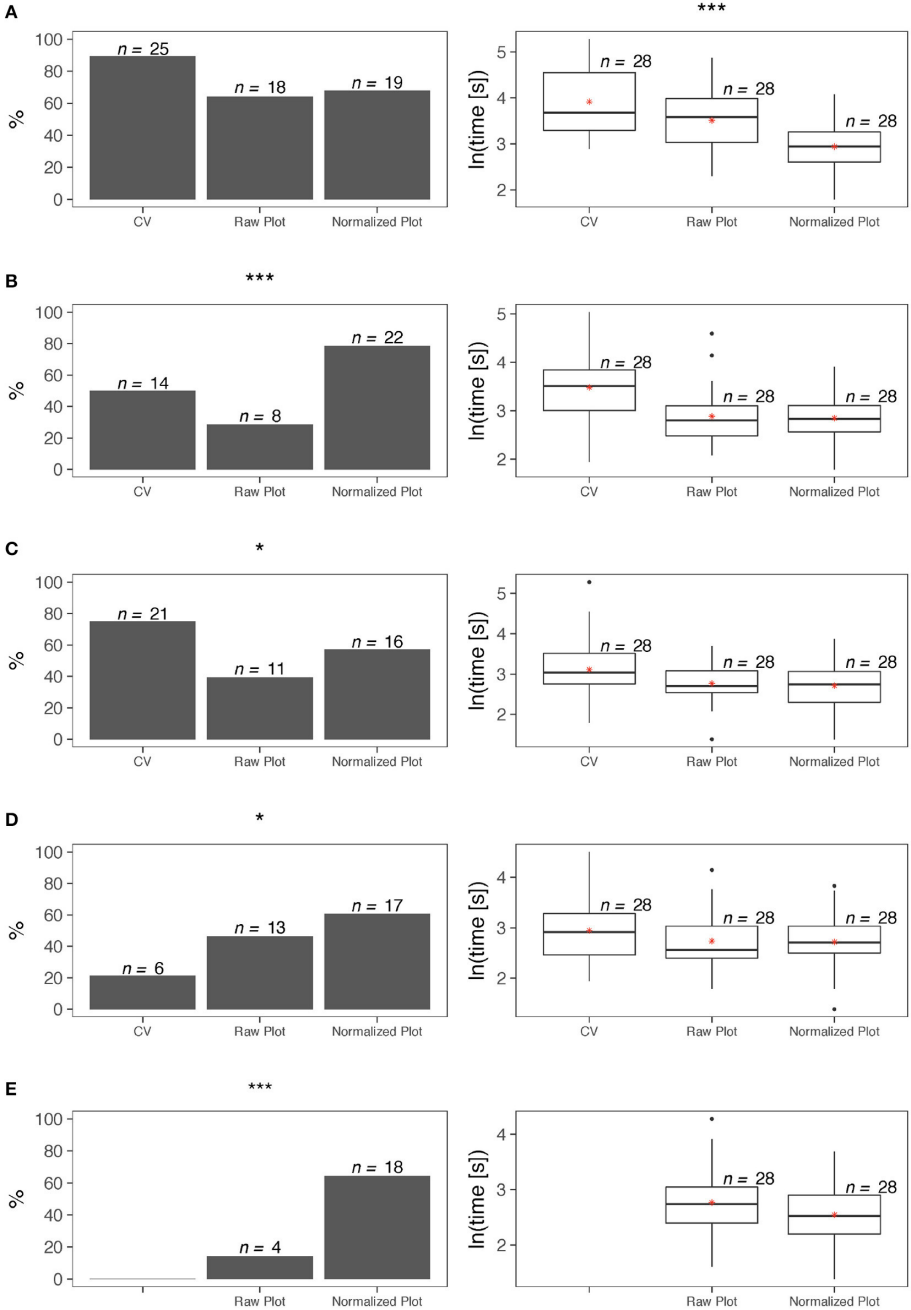
Survey statistics for the plot form of SP–left column depicts accuracy results, while right column depicts completion time results. **(A)** Overall citation record. **(B)** Citation impact of recent publications. **(C)** Overall competitiveness of publications. **(D)** Overall funding record. **(E)** Effect of funding on citation impact. Levels of significance were set at *α = 0.05, **α = 0.01, and ***α = 0.001.

All three forms under examination—CV as Google Scholar profile, SP Raw Plot, SP Normalized Plot - convey quantitative information about citations, although they compute and present this information in different ways. Accordingly, the survey poses the same citation questions across modalities to check if the plots help evaluators to refine their CV-based opinions. In evaluating the overall citation record of a scholar, the respondents did not exhibit significant differences in the accuracy of their assessment, irrespective of the modality they used–CV, Raw Plot, or Normalized Plot (test of proportions, *p* > 0.05). However, they arrived at a conclusion much faster when they used the Normalized Plot (analysis of variance, *p* < 0.001) - Figure 6A.

In evaluating the citation impact of a scholar's most recent publications, the respondents were significantly more accurate in their assessment when they used the Normalized Plot (test of proportions, *p* < 0.001). Moreover, for this item, use of either the Raw Plot or the Normalized Plot facilitated significantly faster decisions with respect to the CV (Kruskal-Wallis, *p* < 0.01) - Figure 6B.

While the SP plots convey quantitative information about the journal impact factors, the Google Scholar profiles do not. Nevertheless, we posed the same question about quality of publications across modalities, because expert evaluators are familiar with the IF of journals in their discipline. Hence, we hypothesized they can form opinions by just reading the journal names in the candidate's CV. The results of the tests confirmed this hypothesis. Indeed, in evaluating the overall competitiveness of a scholar's publications, the respondents were significantly more accurate in their assessment when they used the CV (test of proportions, *p* < 0.05). However, use of either the Raw Plot or the Normalized Plot facilitated significantly faster decisions for this item (analysis of variance, *p* < 0.05) - Figure 6C.

In the case of publication competitiveness, P&T evaluators fill in the information gap in Google Scholar profiles thanks to background knowledge they possess about the standing of journals. In the case of NSF and NIH funding, such a filling in function is more difficult, because there are no apparent indicators in Google Scholar profiles to anchor funding estimations. In focus groups we conducted in the past, however, several P&T evaluators were bragging they could infer if candidates received premium federal funding (i.e., NSF and/or NIH) by just looking at their publication record. The results of the tests rejected this claim. Indeed, in evaluating the overall funding record of a scholar, the respondents were significantly more accurate when they used the Normalized Plot (test of proportions, *p* < 0.05). In terms of time efficiency, there were no significant differences between the three modalities for this item (analysis of variance, *p* > 0.05) - Figure 6D. Although the tests confirmed the value of the Normalized Plot in assessing a scholar's funding record, the claim of some P&T evaluators they can guess funding levels from publication records is not totally unfounded. Indeed, recent research has demonstrated that there is high degree of correlation between the scholars' citation impact and their premium federal funding record (Petersen et al., 2018).

In comparing the cross-factor inferencing power we found that the respondents were far more accurate in assessing the effect of funding on citation impact when they used the Normalized Plot with respect to the Raw Plot (test of proportions, *p* < 0.001); time efficiency did not differ significantly between the two modalities for this item (t-test, *p* > 0.05) - Figure 6E.

#### 3.2.2. Iconic Form of SP—Survey Design and Measurements

Figure 4B depicts the survey design for the iconic form of SP, also known as Academic Garden (AG). The key element of AG is the flower metaphor - a coded picture of a scholar's overall academic merit with respect to citations, competitiveness of publications, and funding. Unlike the plot form, the iconic form lacks an explicit temporal dimension, and thus, it does not prompt inferencing to the same degree the plot form does. Accordingly, the survey questions aimed to merely measure how accurately and quickly the respondents can decipher the flower metaphor.

**Figure 7:**
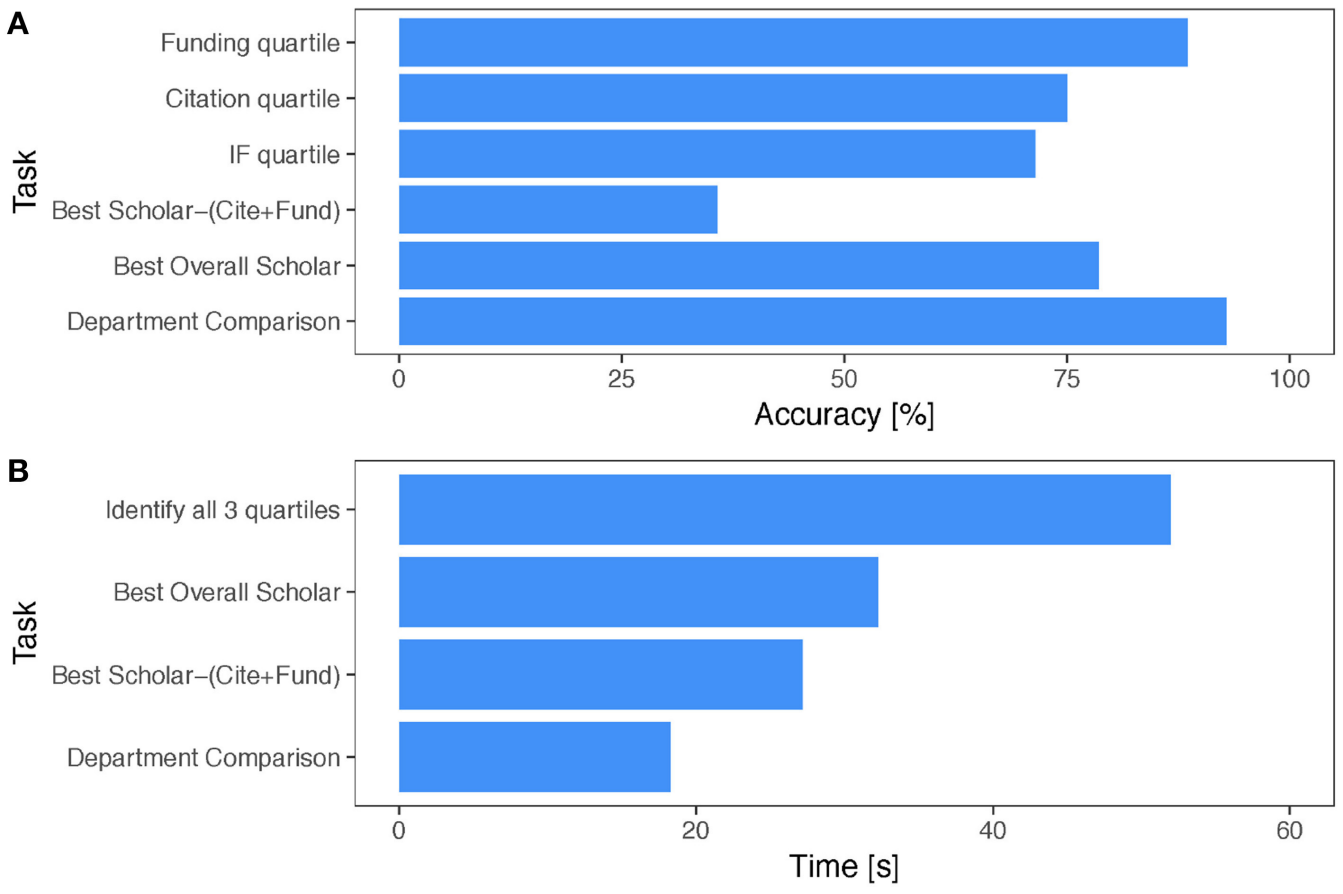
Survey statistics for the iconic form of SP. **(A)** Accuracy scores per task. **(B)** Mean completion times per task.

Looking at the presented flowers in the survey, the respondents were able to decipher the quartiles of the corresponding scholars with respect to citations, publication competitiveness, and funding with an accuracy of 81% and in <60 s (Figure 7A). A question testing the ability of the respondents to compare two departments by looking at their academic gardens yielded even better results - the respondents were able to accurately determine which department was stronger (i.e., had more meritorious faculty) 96% of the time and in <18 s (Figure 7B).

**Figure 8:**
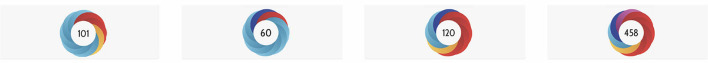
Altmetric badges for various publications. The preponderance of light blue in the two left badges suggests that the corresponding publications drew most of their alternative impact from tweets. The preponderance of red in the two right badges suggests that the corresponding publications drew most of their alternative impact from press coverage. Altmetric badges captured on August 11, 2019 and displayed here with permission from Altmetric.

The survey methodology we applied to evaluate the effectiveness of the flower icon could be generalized in evaluating other iconic forms of scholarly performance. A case in point is the Altmetric badge - a color coded donut for communicating the alternative impact of a publication. Each string color corresponds to a different source of Altmetric impact; for example, light blue indicates twitter trending and red indicates press coverage. In the middle of the donut appears the total numeric score, while the relative color composition of the donut conveys how much each source of Altmetric impact contributed to this score (Figure 8). Based on this badge design, it is difficult for the user to ascribe a specific percentage to each contributing Altmetric factor—a point that is likely to come up if one runs a survey similar to ours for Altmetric badges. In contradistinction, the flower design for P&T merit empowers the user to associate specific quartiles with each factor.

#### 3.2.3. Respondent Perceptions

The last segment of the survey had a series of 5-point Likert scale questions, examining the perceptions of the respondents with respect to the plot and iconic forms of SP. The scale was as follows: 1 ≡ Strongly Disagree; 2 ≡ Disagree; 3 ≡ Neutral; 4 ≡ Agree; 5 ≡ Strongly Agree. Hence, larger numbers corresponded to more positive responses. Respondents ranked highly the design and insightfulness of both forms (mean 3.9). With respect to the plot form, the respondents were highly commendable about the self-awareness it precipitates (mean 3.9). The plot form also received high praise for being easy to understand and easy to use (mean 3.8), while the iconic form received high praise for its visual appeal (mean 3.8). No item had mean below 3.4, which suggests a positive reception from the user sample.

##### 3.2.3.1. Free form feedback

The perceptions segment of the survey ended with a free-form feedback question, inviting the respondents to provide suggestions for improvement. Regarding the plot form, there were three major feedback trends, with the respondents suggesting to: (a) reveal details of the dataset used in the plot normalization; (b) include conference ranking as an additional indicator of publication competitiveness; (c) expand the list of funding sources beyond NSF and NIH.

Regarding the iconic form, there were three major feedback trends, with the respondents suggesting to: (a) simplify the petal color design; (b) simplify the pollinator design; (c) enhance the visual metaphor with a hint about the scholar's academic age (i.e., junior vs. senior faculty).

#### 3.2.4. Iterative Process

##### 3.2.4.1. Improvements in the plot form of SP

**Figure 9:**
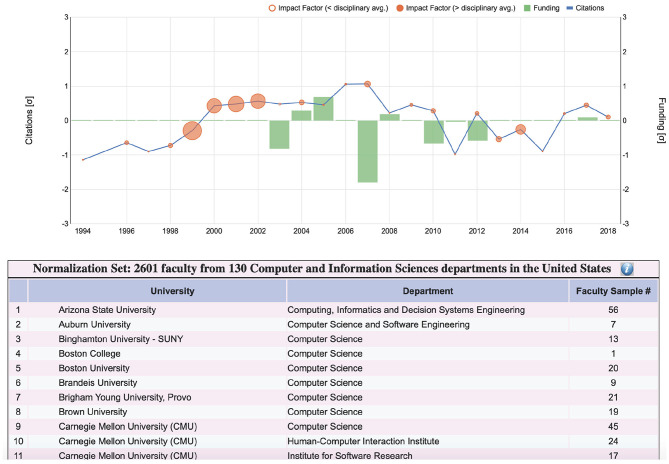
Details of disciplinary sample used in the normalization equation for a computer science faculty whose SP plot is shown at the top. The departments partaking in the sample are listed alphabetically, complete with the corresponding faculty numbers in the database. Due to lack of space, only the first 11 out of 130 computer science departments are shown in this figure. In the actual SP interface, the user can scroll though the entire list.

Following up on the user feedback for the plot form, we have already addressed the first issue - the revised SP features under the plots a statement with the numbers of department and faculty partaking in the normalization calculations. If the user craves for more details, s/he can press the information button at the end of the statement, unveiling a table with the named list of all departments and the power of the faculty sample in each case (Figure 9). With respect to the second issue, we plan to incorporate somehow conference ranking into the plot and iconic forms of SP, after we study them in more detail. As far as the third issue is concerned, SP depends on the publicly available grant tables from research funding agencies. Thankfully, more agencies have recently made available their grant data in the Federal RePORTER, including CDC and NASA; these new funding entries have been incorporated into the revised SP.

##### 3.2.4.2. Improvements in the iconic form of SP

Following up on the user feedback for the iconic form, we addressed all three issues raised with respect to the original flower metaphor—Figure 1 shows the revised AG design to be compared with the older design shown in Figure 3. Specifically, we restricted the petal rounds to alternate between two colors only - pink and white. This makes it very easy to count petal rounds, which code quartiles of publication competitiveness. The resulting flowers also look more natural as they are reminiscent of a particular variety of chrysanthemum. With respect to the pollinator design, instead of having layers of pollinators coding the quartiles of citations, we restricted the pollinator depiction to one layer only, featuring at most four pollinators; each one of these four pollinators counts for one quartile. Finally, to give a hint of the scholar's academic age, we introduced the bud feature in the flower metaphor. Scholars who have academic age less than or equal to 15 years are depicted as buds, while scholars who have academic age greater than 15 years are depicted as fully blossomed flowers. We chose 15 years to be the threshold between junior and senior scholars, by computing the mean academic age at which faculty become full professors on a ground-truthed subset of our dataset. Figure 1 shows a department with two junior faculty, which are depicted as flower buds and seven senior faculty, which are depicted as fully bloomed flowers.

## 4. Discussion

The authors, leveraging their experience with the P&T system in U.S. academia, designed and implemented an open academic metric interface that is simple, insightful, and applicable to both individuals and departments. This interface is known as Scholar Plot (SP) and is meant to complement the conventional academic CV through its plot forms, while generating academic badges through its iconic forms. A multi-stage survey, conceived to test whether SP achieved its objectives or not, was disseminated to STEM faculty in various U.S. universities; P&T evaluation record was a participation requirement. Analysis of responses from *n* = 28 volunteers who completed the survey suggests that SP fulfills its design premise and can enhance critical academic processes.

Specifically, the Normalized Plot of SP enables accurate inferencing about the recent scholarly record of faculty, addressing the temporal right-censoring bias that skews the comparison of citation counts. The Normalized Plot also facilitates evaluation of the faculty's funding record and its apparent effect on his/her citation impact. These are important academic merit criteria that cannot be easily and accurately assessed from conventional CVs alone. Moreover, SP is highly efficient, enabling users to arrive at conclusions very fast.

With respect to SP's plot forms, assessment of publication competitiveness is the key concern that emerges from the survey results. This was the only case where the respondents were on average more accurate when using the CV with respect to SP. Publication competitiveness is the third dimension of the SP plot, realized via disk size. It appears that this visualization choice is confusing to certain users and needs to be re-designed in the future.

With respect to the iconic forms of SP, the respondents quickly and accurately deciphered the flower metaphors and were pleased with the aesthetics of the academic garden. It was particularly impressive the speed with which the respondents were able to compare departments based on their gardens. It appears the more complicated the comparison (i.e., involving multiple units on each side) the easier it becomes, thanks to the general impression conveyed through the garden representation.

Initial thoughts to give academic gardens a randomly colorful look (Figure 3) were not welcomed by the respondents. Consequently, this initial design of the flower metaphor was rolled back in the iteration phase, opting for a totally consistent and simplified iconic coding (Figure 1). The moral is that strict standardization trumps other concerns in iconic interfaces of highly quantitative variables.

Team science is becoming increasingly dominant in scientific research (Wuchty et al., 2007) and the typical number of authors in papers grows larger as time passes by Pavlidis et al. (2014). Hence, the question of how to assign individual credit with respect to citations, venue competitiveness, and research funding becomes critical. In the current version of SP, we assign full credit to all coauthors (or coinvestigators in the case of grants). We avoid fractional credit schemes because are more complicated without bringing the benefit of robust solutions. In more detail, for the issue of authorship credit:

- **Equal fractional credit:** Under such a credit scheme, collaborative authors would see a near uniform reduction to their credit, but solo authors would hold their ground, thus becoming more prominent. This scheme discounts the added value associated with team work and would be objectionable in many scholarly circles.
- **Unequal fractional credit:** Assigning credit anisotropically (e.g., first authors get more credit than other authors) would again benefit solo authors, but would also benefit first authors in team efforts. Such a scheme would not be without controversy either, as sometimes who becomes first author has to do with internal team dynamics rather than pure merit.

In a future version of SP, one way out of this conundrum would be to report credit according to multiple schemes. In the long run, and as more journals adopt the contributorship model (Da Silva, 2011), a better solution would be to annotate each paper with the author's specific contribution (e.g., “author A designed research”), thus turning credit into a multinomial variable.

In closing, we feel compelled to reiterate that quantification and visualization of performance metrics in academia is no substitute for thoughtful and holistic peer evaluation. Such measures have to be used with caution and for the purpose of enhancing information in conventional sources (e.g., CVs). SP is an effort in this direction, focusing on three merit factors that weigh in academic careers and departmental ranking. Our ongoing efforts are directed in expanding the number of modeled factors in SP, while adding diverse methods to compute the three existing factors. For the former, the addition of Altmetrics is in our immediate plans, while for the latter, we are working to add fractional credit and contributorship model schemes.

## Data Availability Statement

The data and their visualization are accessible at Scholar Plot: http://scholarplot.org.

## Author Contributions

DM collected data, developed software, conducted survey, and performed analytics. EA designed graphics. MA developed software and conducted survey. AP developed methods. BU edited manuscript. IP designed research, developed methods, and wrote manuscript.

### Conflict of Interest

The authors declare that the research was conducted in the absence of any commercial or financial relationships that could be construed as a potential conflict of interest.
